# Atypical Localization and Dissociation between Glucose Uptake and Amyloid Deposition in Cognitively Normal APOE*E4 Homozygotic Elders Compared with Patients with Late-Onset Alzheimer’s Disease

**DOI:** 10.1523/ENEURO.0396-17.2018

**Published:** 2018-02-28

**Authors:** José V. Pardo, Joel T. Lee, for the Alzheimer’s Disease Neuroimaging Initiative

**Affiliations:** 1 Cognitive Neuroimaging Unit, Mental Health Service Line, Minneapolis Veterans Health Care System, Minneapolis, MN 55417; 2 Department of Psychiatry, University of Minnesota, Minneapolis, MN 55455

**Keywords:** Aging, Alzheimer’s Disease Neuroimaging Initiative, amyloid, APOE*E4, brain metabolism, positron emission tomography

## Abstract

Alzheimer’s disease (AD) progresses insidiously over decades. Therefore, study of preclinical AD is critical to identify early pathophysiological changes as potential targets for prevention or treatment. The brain processes at the preclinical stage remain minimally understood. Aside from age, the E4 allele of APOE flags a group at particularly high risk of late-onset AD (LOAD). Studies of these individuals could provide insights about the ontogenesis of AD offering clues for novel treatment strategies. To this end, cognitively normal, APOE*E4 homozygotes from the Alzheimer’s Diseases Neuroimaging Research Initiative database (ADNI-LONI) provided fluorodeoxyglucose and amyloid (florbetapir) PET scans (*n* = 8 and 7, respectively; mean age 76 years). Their scans were compared to those of matched cognitively normal elders who were not E4 carriers. There was dissociation in the distribution between glucose uptake and amyloid deposition in the homozygotes. Peak hypometabolism localized bilaterally along the medial temporal cortex. In contrast, peak amyloid deposition localized principally to the putamen, a finding also seen in preclinical carriers of autosomal dominant AD mutations and preclinical AD associated with Down syndrome. Additional regions of amyloid deposition in homozygotes were medial prefrontal cortices including the anterior cingulate, middle and inferior frontal cortices, and middle and inferior occipital cortices. These findings contrast with those reported for LOAD. These data begin to characterize elders with normal cognition despite high AD risk in comparison to the known phenotypes of patients with LOAD.

## Visual Abstract

**Figure:**
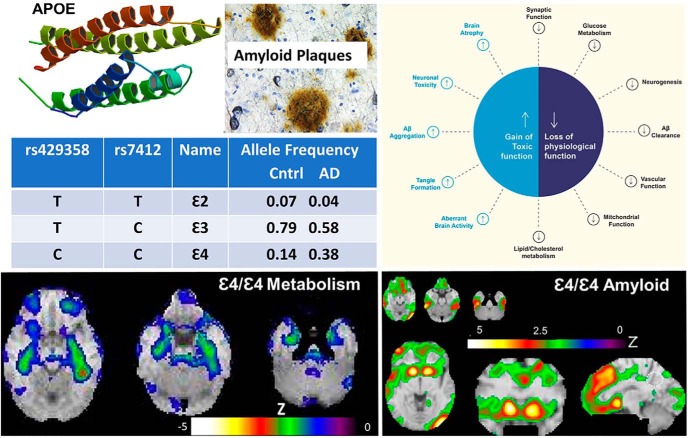

## Significance Statement

APOE*E4 has the largest single effect size of any common variant for any human disease. APOE*E4 homozygotes increase the risk of late-onset Alzheimer’s disease (LOAD) 15-fold. Research indicates that interventions for AD must occur decades before onset of symptoms. However, the phenotypic antecedents and pathophysiology of LOAD remain limited in characterization. APOE*E4 homozygotes offer a unique opportunity to characterize preclinical AD. Here, neuroimaging of cognitively normal, elderly APOE*E4 homozygotes reveals decreased medial temporal metabolism and increased lenticular amyloid deposition in those at high risk for developing LOAD. In comparison to LOAD, an atypical pattern of change in metabolism and amyloid distribution as well as a dissociation between these two measures arose in homozygotes compared to noncarriers.

## Introduction

The disease process of Alzheimer’s disease (AD) begins insidiously decades before enough brain damage occurs to require accessing medical care. Increasingly, there is consensus that prevention is critical and that interventions will be best when used early. The two most significant risk factors for AD are age and the APOE*E4 gene (also referred to as E4).

Having two APOE*E4 alleles gives an odds ratio for AD of almost 15 (Farrer et al., 1997). APOE*E4 is the common variant with the greatest known effect size for association to any human disease. This level of risk is like that for known Mendelian disease-causing mutations such as the breast cancer gene, *BRACA* (Genin et al., 2011). E4 homozygotes have earlier onset of cognitive decline by ∼5–7 years (Blacker et al., 1997; Sando et al., 2008). Therefore, studies of carriers of APOE*E4 have the potential to reveal insights into the early pathophysiology of AD. Recent advances in technology offer unique opportunities to study these individuals noninvasively.

Early in the development of AD, amorphous amyloid deposits occur throughout the brain initially in the inferior aspects of the frontal, temporal, and occipital lobes, later spreading diffusely throughout the neocortex (Braak and Braak, 1991). Amyloid deposition in the form of neuritic plaques containing the amyloid-β (Aβ) protein develop with variable consistency, density, and distribution. Tangles and neuropil threads generally develop before plaques. The presence of APOE*E4 is associated with amyloid deposition even in cognitively normal elders (Morris et al., 2010). The amount and distribution of neuritic plaques vary widely between individuals at similar disease stages (Braak and Braak, 1991). As many as one-third of E4 noncarriers diagnosed clinically with AD are characterized as amyloid negative on neuropathologic assessment (Monsell et al., 2015). In that study, approximately one-half of those with a primary diagnosis of mild to moderate AD and low cerebral Aβ had extensive neurofibrillary degeneration.

The amount of fibrillar amyloid throughout the brain identifies both past and future decline in cognition (Doraiswamy et al., 2014; Donohue et al., 2017). PET studies of AD indicate that fibrillar amyloid and hypometabolism arise early in the posterior cingulate cortex/precuneus (Minoshima et al., 1994, 1997; Scheff et al., 2015). Although there is often overlap between cortical thinning, amyloid deposition, and hypometabolism, dissociations are not unusual (Murray et al., 2014).

Abnormal tau begins to deposit in the transentorrhinal cortex even in nondemented individuals (Braak and Braak, 1991; Bouras et al., 1994; Braak and Del Tredici, 2015). Subsequently, tau spreads to limbic allocortices including the hippocampal, cingulate, retrosplenial, and orbitofrontal cortices. With advanced AD, tau spreads into the neocortex. Tau staging correlates best with level of cognitive function and appears consistent across individuals with similar symptoms and degrees of clinical dysfunction (Braak and Braak, 1991; Ossenkoppele et al., 2016). APOE*E4 status and tau pathology are not correlated (Morris et al., 2010). Although there is frequent overlap between amyloid, atrophy, and tau, dissociation between markers can occur; tau and atrophy tend to co-occur more frequently (Xia et al., 2017).

Biomarker development has advanced greatly the characterization of preclinical and early AD. As many as 30% of cognitively normal individuals >65 years of age have significant amyloid deposition (Murray et al., 2014). PET imaging has demonstrated amyloid deposition in both cognitively intact and impaired carriers of mutations in PSEN1, PSEN2, and APP, who typically have early onset of AD (Klunk et al., 2007; Remes et al., 2008; Fleisher et al., 2012; Shi et al., 2015; Rodriguez-Vieitez et al., 2016). Similarly, amyloid deposition has been found in those with and without evidence of cognitive decline who have Down syndrome (trisomy 21) with development of AD during middle age (Handen et al., 2012; Lao et al., 2016; Rafii et al., 2017).

Cognitively normal healthy elders homozygous for APOE*E4 and therefore at very high risk of developing AD could provide data relevant to preclinical AD. Such information can guide future research and hint at pathophysiological mechanisms, particularly given recent evidence from transgenic mice that APOE*E4 may provide a gain-of-toxic-function independently of amyloid (Shi et al., 2017). To this end, PET scans of glucose uptake with [^18^F]fluorodeoxyglucose (FDG), a proxy for regional cerebral metabolism, and of amyloid deposition from [^18^F]florbetapir scans were downloaded from the Alzheimer’s Disease Neuroimaging Initiative (ADNI) database. Scans from E4 homozygotes were compared to scans from APOE*E4 noncarriers.

## Subjects and Methods

### Participants

Data used in the preparation of this article were obtained from the Alzheimer’s Disease Neuroimaging Initiative (ADNI) database (adni.loni.usc.edu; RRID:SCR_003007; accessed May 2017). The ADNI was launched in 2003 as a public–private partnership, led by Principal Investigator Michael W. Weiner, MD. The primary goal of ADNI has been to test whether serial magnetic resonance imaging (MRI), positron emission tomography (PET), other biological markers, and clinical and neuropsychological assessment can be combined to measure the progression of mild cognitive impairment (MCI) and early AD.

All E4/E4 subjects (*n* = 8) included here had no memory complaints or functional impairments and were identified as normal cognitively based on neurologic and neuropsychological testing; Mini-Mental Status Exam (MMSE) score 24–30; absence of clinically significant findings on screening MRI; Clinical Dementia Rating (CDR) 0; Geriatric Depression Scale (GDS) ≤6; and Hachinski score <4. This designation was based on the diagnosis closest to the time of the first imaging scan. Imaging results (e.g., cortical thinning, FDG, amyloid) were not used to define normality, so normal volunteers could have abnormalities in imaging scans subsequently. One homozygote did not get an amyloid scan. The comparison group was likewise characterized as normal but lacked E4 carriers. Demographic characteristics and related data of the homozygotes are shown in Table 1.

**Table 1.** Subject demographics and related data

FDG ID #	AV45 ID #	Age (y)	Sex	MMSE	Hach	GDS	SUVR	Months	Dx
009_S_4388	009_S_4388	67	M	29	1	2	0.97	0.9	MCI
013_S_4580	013_S_4580	70	F	30	1	2	1.03	24	Nl
014_S_0520	NA	82	F	30	0	1		8.8	MCI
014_S_4577	014_S_4577	85	M	29	1	0	1.17*	0.8^†^	MCI
027_S_5083	027_S_5083	74	M	28	0	1	1.05	24	Nl
032_S_4348	032_S_4348	66	F	30	0	5	1.42*	7.2	Nl
033_S_4179	033_S_4179	83	M	30	1	1	1.53*	52	Nl
082_S_4339	082_S_4339	84	M	29	1	0	1.41*	25	Nl

Reference group matched for age (mean 75 years, range 60–94, SD 6) and education (mean 17 years, range 8–20). Months denote time interval between closest assessment and FDG PET (months to last diagnosis of normal after imaging; ^†^months to last diagnosis of normal before imaging). *, amyloid positive (per ADNI). AV45, florbetapir; MMSE, Mini-Mental Status Exam score; Hach, Hachinski score; GDS, Geriatric Depression Scale; SUVR, standardized uptake value ratio (per ADNI); NA, scan not available/done; Nl, normal; MCI, mild cognitive impairment; Dx, last recorded diagnosis.

Additionally, late-onset AD (LOAD) subjects (*n* = 8) from ADNI with very mild AD (CDR sum of boxes mean = 3.4; range 2–4.5; SD = 1) and similar ages to the E4/E4 group were selected as a patient group for comparison to the observations on asymptomatic homozygotes. As reviewed in the Discussion, there is already an extensive convergent literature on metabolic changes in AD.

### Methods

All methods are described in detail at the ADNI database (adni.loni.usc.edu) following procedures approved by the institutional review boards. All scanning manufacturer’s corrections were “On” (decay, randoms, scatter, etc.).

Briefly, subjects were injected intravenously with 185 MBq (5 mCi) [^18^F]FDG for the glucose-uptake scan. The volunteer rested with eyes and ears open in a quiet, dim room for 20 min. Thirty minutes after injection, emission scans were obtained for 30 min. Scans were corrected for measured attenuation using low-dose CT scans.

Amyloid scans were acquired within 2 wk before or after the FDG scans. The subjects were injected intravenously with 370 MBq (10 mCi) [^18^F]florbetapir. After a 50-min uptake period, an emission scan was obtained for 20 min. Scans were corrected for measured attenuation using low-dose CT scans.

### Image analysis

All scans consisted of a 160 × 160 × 96 image grid with a voxel size of 1.5-mm cubic voxels. Scanner-specific filters were used to obtain an image resolution of ∼8 mm full width at half-maximum (FWHM) regardless of scanner model. Images were inspected visually for potential artifacts. The FDG PET scans were normalized to a whole-brain uptake of 1000 counts. The amyloid scans were normalized based on the cerebellar cortex. All scans were anatomically coregistered to a template using Neurostat (Stereotactic Image Registration; v.7.1; S. Minoshima, University of Utah). Minima and maxima of the *z*-images were localized and quantitated with in-house software using an averaged, roving, 3-voxel cube. As routine for FDG PET studies at this resolution, a *z*-score ≥3.0 was defined as significant. The localization of structures was aided through use of the Talairach daemon (Lancaster et al., 2000).

## Results

The demographics and related data of the APOE*E4 homozygotes are presented in Table 1. The average age was 76 y, range 66–85, SD 8. Three volunteers were women. The average of MMSE scores was 29. SUVR ranged from 0.93 to 1.53 (mean 1.02; SD 0.04); four were classified as amyloid positive by ADNI criteria. Hachinski scores ranged from 0 to 1 with average of 0.5. The average GDS rating was 1.5. The reference control group (i.e., non-E4 carriers; including E2 and E3 genotypes) was matched for age (75 y old, range 60-94, SD 6) and educational level (17 y). The reference group for FDG PET included 282 subjects; 144 males, 138 females; education range 8–20 years, mean = 16.6; 254 European Americans, 17 African Americans; 6 Asian Pacific Americans; and 1 Native American. The reference control group for amyloid imaging included 263 subjects; 133 males, 130 females; 235 European Americans, 17 African Americans, 6 Asian Pacific Americans; and 1 Native American.

**Figure 1.**
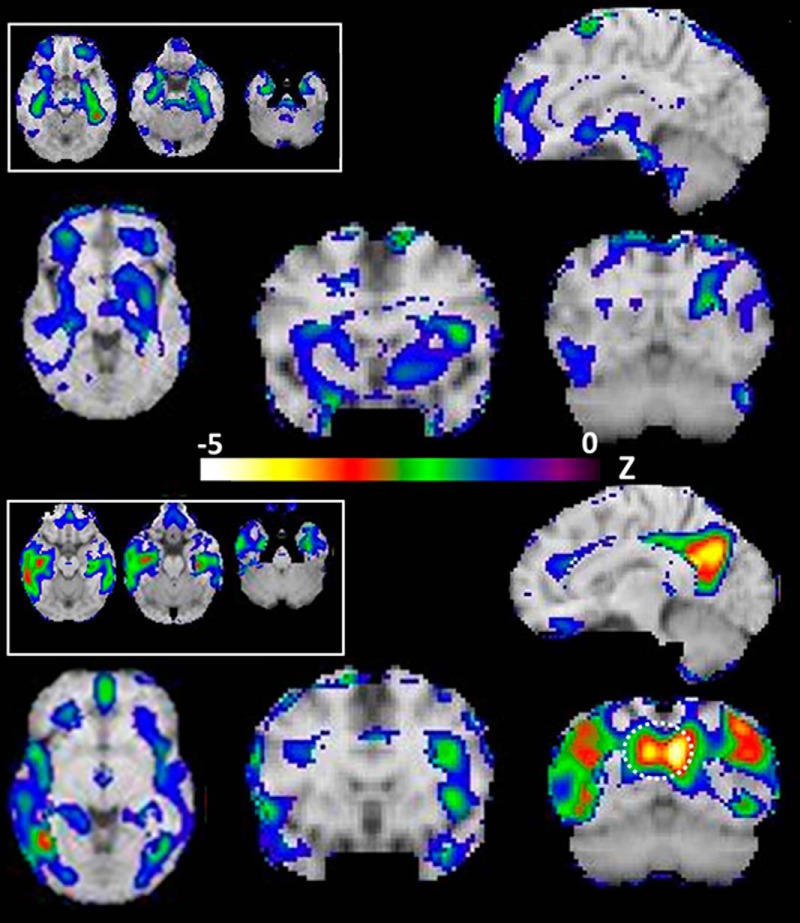
Top (above color bar): FDG uptake in E4 homozygotes contrasted with E4 noncarrier reference group. Transverse sections (top left inset) are taken from left to right at *z* = –11, –18, and –25 mm below the intercommissural plane, respectively. The peak (red; *z* = –3.0) is in the left parahippocampus. Note sequential inferiorly directed medial temporal lobe structures. Larger sections from lower left to upper right: *z* = –2, *y* = 12, *y* = –60, *x* = –10, respectively. Note minimal hypometabolism throughout including striatum. For illustrative purposes, the threshold was set to *z* = –3. Note minimal or no hypoactivity in PCC and parietal cortex in E4 homozygotes. Bottom: FDG uptake in very mild AD contrasted with E4 noncarrier reference group. Transverse sections (top left inset) are taken from left to right at *z* = –11, –18, and –25 mm below the intercommissural plane, respectively. Note sequential inferiorly directed medial temporal lobe structures. Larger sections from lower left to upper right: *z* = –2, *y* = 12, *y* = –60, *x* = –10, respectively. For illustrative purposes, the threshold was set to *z* = –3. Note marked hypometabolism in bilateral lateral parietal cortex and PCC/precuneus (dashed circle). Left side of brain is on right side of image (radiologic convention).

Fig. 1 (top) shows the peak regions of hypometabolism based on [^18^F]FDG when comparing subjects who were APOE*E4 homozygotes versus APOE*E4 noncarriers. Both medial temporal cortices were symmetrically hypometabolic (left greater than right). The peak of hypometabolism mapped to left parahippocampal gyrus, Brodmann 37, at (–39, –37, –11) with *z* = –3.0. Although below the significance threshold, the second most hypometabolic peak localized to the left hippocampus at (–33, –15, –14) with *z* = –2.6. No other regions were hypometabolic including the putamen or ACC. No regions showed increased metabolism in the contrast between homozygotes and noncarriers.

Fig. 1 (bottom) shows the results for the same metabolism contrast (LOAD vs. noncarrier). There is medial temporal hypometabolism similar in magnitude to that seen in E4/E4. As expected, large foci of hypometabolism localized to the PCC, ACC, and lateral parietal and inferior temporal cortices.

**Figure 2.**
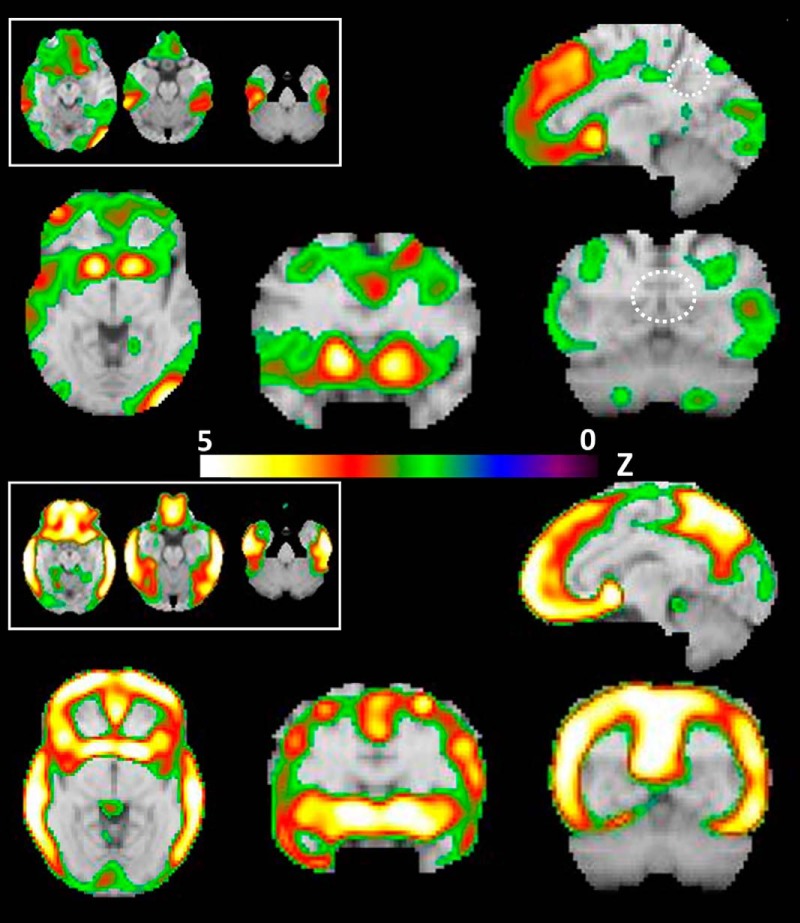
Top (above color bar): Amyloid deposition in cognitively normal, E4 homozygotes contrasted with E4 noncarriers. Transverse sections through MTL (top left inset) are from left to right at *z* = –11, –18, –25, respectively. Larger sections from lower left to upper right: *z* = –2, *y* = 12, *y* = –60, *x* = –10, respectively. Note heavy amyloid deposition in the striatum, specifically the putamen. The PCC/precuneus (dashed circle) appear to have minimal amyloid, unlike the typical pattern in LOAD (see bottom panel). For illustrative purposes, the threshold was set to *z* = –3. Bottom panel: Amyloid deposition in very mild AD contrasted with E4 noncarriers. Transverse sections through MTL (top left inset) are from left to right at *z* = –11, –18, –25, respectively. Larger sections from lower left to upper right: *z* = –2, *y* = 12, *y* = –60, *x* = –10, respectively. Note extensive amyloid deposits throughout the cortex, especially PCC/precuneus, and the striatum. Left side of brain is on right side of image (radiologic convention); color bar indicates *z*-scores.

Fig. 2 (top) shows the loci of peak deposition of amyloid based on [^18^F]florbetapir in APOE*E4 homozygotes when compared to the E4 noncarrier group. The peaks with highest magnitude localized to the lenticular nuclei, particularly the bilateral putamen. A broad region of amyloid deposition occurred in the ACC as well as medial and middle prefrontal gyri, inferior temporal, and occipital gyri. Other foci are listed in Table 2. No amyloid mapped to the PCC.

**Table 2.** Amyloid deposits in cognitively normal E4 homozygotes contrasted with E4 noncarriers

	Peak location^a^			
Structure	*x*	*y*	*z*	*z*-score
Putamen	15	12	0	4.7
Inferior occipital Brodmann area 18	–35	–85	–5	4.6
Putamen	–15	12	–2	4.5
Middle occipital Brodmann area 19	–44	–73	–9	4.2
Inferior temporal Brodmann area 20	55	–37	–18	4.1
ACC Brodmann area 32	–6	21	36	4.0
ACC Brodmann area 32	–8	23	45	4.0
Middle frontal Brodmann area 8	28	26	47	4.0
Brodmann area 9	28	41	29	3.9
Inferior temporal Brodmann area 20	39	–24	–23	3.8
Middle frontal Brodmann area 10	42	50	0	3.7
Medial frontal Brodmann area 9	–10	44	20	3.6
Middle frontal Brodmann area 8	–24	35	43	3.6
Brodmann area 9	–28	46	27	3.5
Brodmann area 36	–48	–40	–20	3.4
Brodmann area 6	–24	8	56	3.3
Inferior temporal BA 20	–46	–10	–27	3.3
Inferior temporal BA 20	–48	–17	–25	3.2
Brodmann area 10	–10	35	–13	3.2
Superior temporal Brodmann area 22	53	1	–2	3.2
Inferior temporal Brodmann area 20	35	–1	–34	3.1
Premotor Brodmann area 6	28	–13	58	3.1
Middle temporal Brodmann area 21	62	–19	–7	3.1

Locations in Talairach (Talairach and Tournoux, 1988) coordinates (mm): +*x*, right; –*x*, left; +*y*, anterior; +*z*, superior. ACC, anterior cingulate cortex.

Fig. 2 (bottom) shows the results for the same amyloid contrast (LOAD vs. noncarrier). Most of the cortex shows extensive deposition of amyloid. The bilateral putamen show the greatest magnitude of deposition along with very heavy deposits in PCC/precuneus, ACC, as well as prefrontal, lateral parietal, and lateral temporal cortices. As reviewed below, these results converge with extensive literature but are presented for direct comparison with the homozygotes.

## Discussion

### Novel findings in E4 homozygotes

This report provides several new findings. The literature relevant to these results based on metabolic and amyloid imaging as well as limitations is presented in the following sections.

(1) Medial temporal lobe (MTL) metabolism was reduced in E4 homozygotes, like the results found here in LOAD, albeit the latter had more extensive lateral temporal changes. Nevertheless, reports of MTL metabolism in relation to E4 status have been mixed. Although cognitively intact E4 homozygotes are widely considered to show patterns of hypometabolism similar to those of patients with early LOAD, there are also numerous regions where they differ; e.g., E4 homozygotes show more extensive hypometabolism in the prefrontal cortex than those with early AD.

(2) E4 homozygotes did not show the anticipated parietal hypometabolism found by others, as reviewed below. Examining all hypometabolic foci, a PCC region occurs at *z* = –2.0, far below the significance cutoff, which could be a type II error given the small sample size. However, similar sample sizes in MCI or AD show robust PCC hypometabolism as seen in LOAD here. The E4 homozygotes in this study were older than in other studies. The absence of parietal hypometabolism could reflect a degree of resilience against AD despite carrying the risk alleles (i.e., sampling bias).

(3) E4 homozygotes showed the greatest degree of amyloid deposition in bilateral putamen. Numerous studies as noted below have shown that the PCC/precuneus and prefrontal cortices have high amyloid loads in cognitively normal adults, early-onset AD, LOAD, and those carrying AD-relevant mutations. However, mutation carriers show highest amyloid deposition in striatum.

(4) The E4 homozygotes showed no hypometabolism in the putamen, the principal site of amyloid deposition. This is consistent with studies of preclinical and clinical carriers of AD-relevant mutations.

(5) Extensive accumulation of amyloid in E4 homozygotes localized to several additional regions including the ACC, medial frontal, middle frontal, inferior temporal, middle temporal, superior temporal, and occipital regions.

### Hypometabolism in AD, E4, and autosomal dominant carriers

#### Metabolic changes in AD

FDG PET remains among the best methods to evaluate functional brain decline in cognitively intact E4 carriers as well as in both preclinical and clinical early-onset AD (EOAD) and sporadic LOAD. EOAD is classified typically as onset before age 65 y. Many studies of EOAD do not systematically screen to exclude carriers of known mutations. Across studies, the greatest consistency and degree of hypometabolism localizes to the PCC/precuneus (see below). It was unanticipated that the MTL was not the principal region of hypometabolism given its role in memory, site of tau deposition, and atrophy in normal aging and AD. However, the large partial volume effects and interslice distances in early PET scanners likely reduced sensitivity to detection.

Kim et al. (2005) studied 74 EOAD (<65 y; CDR 0.5) using FDG PET. They reported more severe hypometabolism in parietal, frontal, and subcortical (basal ganglia and thalamus) areas compared with LOAD, interpreted as a more rapid deteriorating course. Rabinovici et al. (2010) reported EOAD (<65 y) had more severe deficits in working memory and attention. EOAD had more severe PCC and bilateral temporoparietal hypometabolism than LOAD; LOAD did not show relative decreases in metabolism compared to EOAD. EOAD had lower metabolism after atrophy correction than LOAD in bilateral precuneus and right angular gyrus; no regions in LOAD were less metabolic than in EOAD. There was a positive correlation between age of onset and hypometabolism in the precuneus, lateral parietal, and occipital regions.

The initial region of hypometabolism in early LOAD localized typically to the PCC/precuneus with subsequent extension into biparietal and inferior temporal regions (Minoshima et al., 1994, 1997). Despite the noteworthy absence of MTL hypometabolism in early studies, most subsequent studies using FDG PET on higher resolution scanners or with interleaf acquisition demonstrated hypometabolism in the MTL along the continuum from normal to MCI to AD; this hypometabolism predicted subsequent cognitive decline (Mosconi et al., 2008; Chen et al., 2010; Lehmann et al., 2014).

#### Metabolic changes with carriage of E4 allele

Several studies examined alterations in metabolism in presymptomatic subjects and LOAD relative to E4 allele status. Small et al. (1995) compared FDG PET scans from cognitively intact subjects (age ∼55 y) with mild memory complaints both with (*n* = 12) and without (*n* = 19) the E4 allele. They found that E4 carriers had marked hypometabolism in both parietal lobes. Reiman et al. (2001) compared metabolic decline over a 2-y interval in cognitively normal APOE carriers versus noncarriers (50–63 y). They demonstrated, despite the absence of cognitive change during follow-up, significantly less metabolism in carriers localized to lateral temporal cortex, PCC, lateral prefrontal cortex, basal forebrain, parahippocampal/lingual gyri, and thalamus. Langbaum et al. (2009) compared FDG PET scans from elder normal control subjects and patients with amnestic MCI and AD. Despite higher resolution (8 mm FWHM) scans, medial temporal hypometabolism was detected bilaterally only in the AD patients compared with control subjects. There was hypometabolism in bilateral precuneus and left lateral parietal lobe in normal subjects with E4 allele compared with those without E4 (*n* = 21 and 61 in each group, respectively).

In a landmark publication, Reiman et al. (1996) contrasted (1) FDG PET of 11 cognitively intact E4 homozygotes with 22 healthy controls without the E4 allele (mean group age: 55 and 56 y, respectively); and (2) FDG PET of 37 probable AD patients with 22 healthy controls (each group of average 64 y old). The probable AD patients relative to matched controls showed three broad regions of hypometabolism: PCC/precuneus as well as bilateral parietal and bilateral inferior temporal lobes. In addition, there were several small foci of hypometabolism in the prefrontal, occipital, and lateral temporal regions. Medial temporal hypometabolism was not reported in the AD patients. In the contrast involving E4 homozygotes versus those without E4 allele, several hypometabolic foci converged with the AD hypometabolic regions, including the PCC/precuneus, parietal, and inferior temporal cortices. Of note, the E4 homozygotes also had broad regions of significant hypometabolism in the prefrontal cortices, as well as smaller foci distributed throughout the cortex and cerebellum, not seen in the AD group.

In a subsequent study, Reiman et al. (2004) used FDG PET to examine healthy middle-aged adults (20–39 y). Twelve E4 heterozygotes compared with 15 E4 noncarriers showed some overlap with hypometabolic regions seen in AD, particularly in PCC/precuneus as well as in parietal and inferior temporal regions. Medial temporal changes were not reported. The heterozygotes showed several regions of hypometabolism in prefrontal cortex beyond those seen in AD patients. So, hypometabolism can arise both within and outside AD-affected regions, even in much younger healthy subjects.

De Leon et al. (2001) noted in a longitudinal study that those having cognitive decline from normal status at baseline showed lateral temporal lobe hypometabolism dependent on E4 status; the entorhinal cortex did not show this effect. Mosconi et al. (2004) compared using FDG PET AD patients with and without the E4 allele. They noted an age-by-genotype interaction in the anterior cingulate and medial frontal cortices. The results were interpreted as indicating an age-dependent aggravation in metabolic decline in AD related to the E4 allele status.

To investigate the effects of ethnicity on the relationship of APOE status to glucose metabolism, cognitively intact, middle-aged (mean ∼55 y) Latino Mexican-Americans were studied with FDG PET (Langbaum et al., 2010). The left hippocampus had decreased metabolism in E4 carriers versus noncarriers (*n* = 11 and 16 per group, respectively). There was some convergence of medial and lateral parietal hypometabolism in the contrast between E4 carriers versus noncarriers with the hypometabolism seen in LOAD. However, the E4 carriers showed less hypometabolism in the traditional precuneus/PCC regions with more extensive involvement of the ACC.

In contrast, Protas et al. (2013) examined with defined regions of interest a large series of healthy subjects (mean age ∼56 y) who carried either 0, 1, or 2 E4 alleles (*n* = 76, 42, and 31, respectively). Those authors noted a highly significant difference across groups in PCC metabolism; no difference in hippocampal metabolism or volume was found. Differences in subject groups, technologies, or analysis methods could account for the divergence in results from those reported here. First, the much smaller sample of homozygotes in the present study compared to that of Protas et al. (2013) could explain the failure to detect PCC hypometabolism; even the changes in the MTL reported here were not large, although there was some subthreshold hypometabolism in the contralateral hippocampus. The left hippocampal hypometabolism in normal E4 carriers reported here does converge with the observation of Langbaum et al. (2010). Second, the earlier-generation scanner used by Protas et al. (2013) had an interslice distance of 3.375 mm; this slice thickness without 3D acquisition or volume reconstruction would decrease recovery of the thin strip of MTL extending inferiorly from posterior to anterior as seen in Fig. 1 in the present report. Third, the mean age of the homozygotes here was much greater than that in Protas et al. (2013). Fourth, the present analysis used a voxel-wise approach, whereas Protas et al. (2013) used defined regions of interest. Finally, Protas et al. (2013) did not report amyloid deposition or longitudinal outcomes that could impact metabolism across subjects and studies.

#### Metabolic changes in autosomal dominant mutation carriers

Several studies have examined with FDG PET the metabolic changes in mutation carriers including *APP* dosage effects (trisomy 21/Down syndrome; *APP* duplication; exon deletion variant) as well as mutations in *PSEN1*, *PSEN2*, and *APP*. Villemagne et al. (2009) found that the classic PCC and biparietal pattern of hypometabolism seen in sporadic AD was not as evident in mutation carriers (*n* = 8; CDR 0–3.0). No distinct pattern of FDG hypometabolism characterized the various PSEN1 versus APP mutation carriers; three individuals showed an almost normal pattern of uptake including the striatum, the principal locus of amyloid deposition. There was no relation between the hypometabolic pattern in the cortex or striatum and disease severity, type of mutation, or cognitive status. Schöll et al. (2012) reported on two *APParc* mutation carriers showing AD-typical patterns of hypometabolism. Sabbagh et al. (2015) reported in Down syndrome without dementia (mean age ∼36 y) mostly hypometabolism and decreased gray matter atrophy in the ACC not PCC. Therefore, considerable variability in metabolism is seen in mutation carriers.

### Amyloid deposits in EOAD, LOAD, E4 carriers, and autosomal mutation carriers

#### Amyloid deposits in AD

Neuropathologically, nondemented control subjects do not show amyloid deposition or neurofibrillary changes (Braak and Braak, 1991). In contrast, patients with AD show not only extensive fibrillar amyloid, but also an abundance of amyloid deposits that are diffuse without neurofibrillary changes or glial reaction (Braak and Braak, 1991; Brilliant et al., 1997). The striatum develops senile neuritic plaques and neurofibrillary tangles later in the progression of AD (Braak stage V–VI; Braak and Braak, 1991).

Amyloid imaging labels fibrillar amyloid (Ikomonovic et al., 2008; Clark et al., 2011; Curtis et al., 2015). Other forms of amyloid (diffuse, fleecy, deposits in AD cerebellum, amyloid oligomers) are not detected. In AD, the greatest fibrillar amyloid deposition localizes to the parietal cortex (both precuneus/PCC and lateral parietal); parietal fibrillar amyloid deposition often shows a correlation with declining parietal metabolism and synaptic markers (Klunk et al., 2004; Li et al., 2008; Scheff et al., 2015). Subsequently, fibrillar amyloid deposits spread throughout the neocortex including ACC, lateral prefrontal cortex, striatum, and the temporal lobe. Differences in amyloid deposition between EOAD and LOAD have depended on the region of interest. Rabinovici et al. (2010) studied 18 EOAD and compared them to 16 LOAD. They found no effect on amyloid deposition but decreasing metabolism in posterior brain regions depending on age of onset. Youn et al. (2017) studied nine EOAD, 11 LOAD, and 8 normal controls. EOAD patients were screened negative for the common AD-associated mutations. EOAD patients showed greater amyloid deposition only in the thalamus and basal ganglia compared to those with LOAD.

#### Amyloid deposits and APOE*E4

The major locus of amyloid deposition found here in E4 homozygotes localized to the putamen as well as several other regions (ACC, medial frontal, middle frontal, inferior temporal, middle temporal, superior temporal, and occipital). Unlike what is seen in typical LOAD, neither hypometabolism nor amyloid deposition localized to the PCC in these E4 homozygotes. It is noteworthy that the localization of fibrillar amyloid in healthy elders and in AD does not reflect the patterns of cortical thinning, hypometabolism, or clinical phenotypes; these appear to converge with the localization of tau (Ossenkoppele et al., 2016; Schöll et al., 2016; Xia et al., 2017; Lockhart et al., 2017; Pontecorvo et al., 2017).

The pattern of amyloid deposition noted here is not inconsistent with that reported by Reiman et al. (2009). They studied a younger group (mean age ∼63 y) of 8 homozygotes, 12 heterozygotes, and 12 noncarriers of E4. They reported a significant association with “AD-affected mean cortical, frontal, temporal, posterior cingulate-precuneus, parietal, and basal ganglia ROIs.” Based on an estimation of their basal ganglia data, reanalysis specifically comparing E4 homozygotes to noncarriers, analogous to that done here, produced *p* < 0.01 (two-tailed, between-sample *t* test; df = 18; heteroscedastic correction) indicating convergence in findings. Given the older age of the current homozygotes, amyloid deposition here appeared grossly greater overall than that reported by Reiman et al. (2009).

#### Amyloid deposits in autosomal dominant mutations

Genetic familial effects beyond those associated with the E4 allele have been reported with APP gene dose (trisomy 21/Down syndrome; APP duplication) as well as mutations in PSEN1, PSEN2, and APP. Putaminal amyloid deposits, which can correlate with age early in the course, were seen in Down syndrome without dementia (Handen et al., 2012; Lao et al., 2016). Braak stage (based on tau scans), amyloid deposition, and cognitive decline were correlated with age in amyloid-positive, nondemented subjects with Down syndrome; glucose hypometabolism and tau did not localize to the striatum, although regions with hypometabolism overlapped areas with tau deposition (Rafii et al., 2017). High levels of amyloid in the putamen were reported also in various cases of preclinical and clinical AD related to PSEN1, PSEN2, and APP mutations (Klunk et al., 2007; Remes et al., 2008; Koivunen et al., 2008; Villemagne et al., 2009; Fleisher et al., 2012; Shi et al., 2015; Rodriguez-Vieitez et al., 2016). Like observations here about E4 homozygotes, AD mutation carriers with putaminal amyloid deposits did not show hypometabolism in the putamen (Rodriguez-Vieitez et al., 2016).

The clinical significance of striatal amyloid deposition is unclear. The co-occurrence of Parkinson’s in AD is well known. AD variants with mutations such as APP can also have various motor manifestations such as hyperactive reflexes, extremity weakness, and spastic paraparesis. An increased incidence of Parkinson’s in subjects with Down syndrome with or without dementia remains controversial (Hestnes et al., 1997).

## Limitations

The major limitation of this study is the small number of homozygotes available in the ADNI database, not surprising given their low frequency. The ALZGENE database indicates an E4/E4 prevalence of ∼2% in controls and 15% in AD cases (Alzforum, 2017). Furthermore, the frequency of E4/E4 decreases with aging, most likely owing to differential survival related to cardiovascular disease. In an Australian community sample of those 70 y or older surviving a follow-up period of 3 y, the E4 allele frequency was 13% (Henderson et al., 1995). Of these 638 subjects, only 10 E4 homozygotes were recruited, and none were older than 90 y. Larger samples will require pooling across multiple databases. The limited sample described here risks type II errors; thus, the present findings must be considered preliminary, particularly regarding the absence of changes in various regions. Nevertheless, the present findings are noteworthy given the differences from LOAD and similarities to AD-related mutations and Down syndrome.

Another issue concerns whether the homozygotes represent typical preclinical AD or whether the sample is biased (e.g., survival bias). Despite normal cognitive status, approximately half were already positive for amyloid by the usual criteria (ADNI’s templated SUVR). Their mean age of ∼76 y is near the peak age (60–75 y) for the highest risk of conversion from normal cognition to MCI or AD (Bonham et al., 2016). The predicted age for onset of LOAD in E4 homozygotes has been estimated at 5–7 y earlier than for noncarriers (Blacker et al., 1997; Sando et al., 2008).

No atrophy correction was made for the measurements of glucose metabolism in this study. The between-group comparison minimized age-related atrophy across groups by matching on age. The rationale for not performing atrophy correction considered that depending on the algorithm, amplification of noise compounded by the small sample size can introduce additional concerns. The question of PCC and medial temporal atrophy in cognitively normal E4 carriers is controversial given mixed results from different studies. For example, Li et al. (2016) found greater atrophy of the left hippocampus in E4 carrier versus noncarrier groups without dementia (i.e., control and MCI combined) from ADNI (*n* = 212 and 242 per group, respectively). Haller et al. (2017) studied adults (*n* = 282) dwelling in the community who were cognitively intact and followed for 18 mo. They found a significant effect of the E4 allele on PCC atrophy but only for those who decline on follow-up; no changes were found in the MTL. The MTL in elderly with intact cognition is known to have tau tangles typically associated with neurodegeneration, whether amyloid positive or negative, and may explain the observed hypometabolism found in this study (Bouras et al., 1994; Braak and Del Tredici, 2015).

## Summary

In conclusion, cognitively normal, elderly E4/E4 show an atypical pattern of both hypometabolism and amyloid deposition compared with that known to occur in LOAD. Metabolism is dissociated in localization from amyloid deposition. The region of greatest amyloid deposition localizes to the putamen, as is seen in Down syndrome and early-onset AD arising from mutations. The mechanisms for protein deposition remain unclear. The difference in biomarker phenotypes between E4 homozygotes and those with LOAD suggest divergent pathophysiological processes, unshared environmental effects, or residuals of resilience to AD in a high-risk group.

## Synthesis

Reviewing Editor: Jörg Schulz, RWTH Aachen University Decisions are customarily a result of the Reviewing Editor and the peer reviewers coming together and discussing their recommendations until a consensus is reached. When revisions are invited, a fact-based synthesis statement explaining their decision and outlining what is needed to prepare a revision will be listed below. The following reviewer(s) agreed to reveal their identity: William Klunk, Jorge J. Palop.

This study compares the amyloid deposition patterns (florbetapir) and cerebral glucose metabolism (FDG) in 7-8 cognitively normal APOE4 homozygotes to that in matched cognitively normal elders non-E4 carriers. The manuscript shows that cognitively normal ApoE4 homozygotes have decreased medial temporal metabolism, particularly in the parahippocampal gyrus (Figure 1) and increased amyloid deposition, particularly in the striatum and anterior cingulate cortex (Figure 2). No amyloid was detected in the precuneus. The authors conclude that compared to late-onset Alzheimer's disease, ApoE4 homozygotes have an atypical pattern of hypometabolism and amyloid deposition.

Although the reviewers agreed that there was interest in the study, its novelty, the presentation of data and their interpretation was questioned. Particularly much more attention must be payed to the existing literature:

1. There is insufficient description of the E4 NON-carrier reference group. The average{plus minus}SD values should be included at the bottom of Table 1. How were they matched?

2.Reiman 2009 did not report striatal deposition in E4/E4 homozygotes - as might be concluded according to the description in this manuscript. The Reiman 2009 paper should be more accurately described in the discussion.

3. Although the findings are interesting, similar findings have been previously reported. For example, regarding the hypometabolism in the hippocampus, Lehmann et al., (2014) showed that ApoE4-carriers AD showed greater hypometabolism medial temporal regions, including the hippocampus. It is concerning to see that the authors are not aware of these findings. The authors also missed Langbaum et. al, Arch. Neurol (2010) that shows left hippocampal hypometabolism in cognitively normal ApoE4-carriers. They also did not cite or discuss hallmark publications that contradicts their main findings. For example, Reiman el al., N. Engl J Med, 1996, showed that cognitively normal ApoE4 homozygotes have hypometabolism in the same regions as in patients with probable AD, which has been reproduced many times. The authors cannot conclude that ApoE4 homozygotes have an atypical pattern of hypometabolism because overall the described patterns matched the pattern of AD patients and all the above previous reports. In addition, the authors did not include late-onset AD cases so they cannot established such conclusions without data or ignoring previous reports. Regarding the amyloid in the striatum, the first report using PIB in late-onset AD patients (Klunk, 2004), which is also not cited, identified PIB retention in the striatum.

4. The discussion needs improvement. The authors need to highlight the novelty of the new findings in the context of the previous published data with scientific rigor. Instead, the authors focused on atrophy and tau imaging, which were not studied.
